# Supplementary material for: Intestinal Epithelial Cells Adapt to Chronic Inflammation through Partial Genetic Reprogramming
Source: Cancers (Basel). 2023 Feb 3;15(3):973. doi: 10.3390/cancers15030973 (PMC9913703; doi:10.3390/cancers15030973)
Supplement: Supplementary file 1 [file cancers-15-00973-s001.zip › cancers-2188031-Supplementary File S1.pdf]

Intestinal epithelial cells adapt to chronic inflammation through partial genetic reprogramming.

Guillaume Collin, Jean-Philippe Foy, Nicolas Aznar, Nicolas Rama, Anne Wierinckx, Pierre Saintigny, Alain Puisieux, Stéphane Ansieau.

Supplementary Materiel and Methods

In silico analysis for validation of the experimental OSAP

Raw data from CEL files were processed using quantile normalization and the robust multi-array average (RMA) algorithm, and were log2 transformed[1]. In order to improve the precision and accuracy the probe set estimates used for our analysis[2,3], updated Affymetrix probe set definitions of the CDF file version 18 (released on Jan 23, 2014) available on the BrainArray web database were downloaded. Gene annotation corresponding to the NCBI ("Entrez Gene")[4] was used. Hierarchical Clustering Analysis and Principal Component Analysis (PCA) were performed using expression data in order to provide an overview of the data, especially to visualize the distribution of both samples according to the expression of OSAP. Unsupervised hierarchical clustering using the Pearson correlation and Ward linkage method was computed. The 2 first components of PCA were used. The single-sample GSEA (ssGSEA) projection methodology from GenePattern[5], an extension of GSEA that enabled the computing of separate enrichment scores for sample/signature pair (e.g., tumor versus normal), was also used. The gene expression values for a given sample were rank-normalized, and an enrichment score was produced using the Empirical Cumulative Distribution Functions (ECDF) of the genes in the signature and the remaining genes[6,7]. Before running ssGSEA, we used the "Collapse Dataset" module from GenePAttern, in order to collapse all probe set

expression values for a given gene into a single expression value (according to the median). Using the human expression datasets downloaded from public repositories, the OSAP scores were calculated for each sample. The immune signature[8] and the two intestinal stem cell signatures Lgr5-GFP^{high} and EphB2^{high} [9] were similarly scored in each sample and the correlation between these signatures and the assessed OSAP was assessed using a Spearman correlation. The Spearman Correlation Coefficient (r) was also estimated to measure the strength of an association between two continuous variables. The Spearman correlation coefficient r can take a range of values from +1 (positive association) to -1 (negative association). Stem-like signature was defined on the basis of the 177 genes enriched in the Stem-like (expression value >0.2) subgroup with respect to the inflammatory, goblet-like, enterocyte and TA molecular subgroups.

Nuclear extractions

Cells were washed with 1X PBS and resuspended in 200 µL of a 10 mM Hepes pH 7.5, 10 mM KCl, 0.1 mM EDTA, 0.5% NP40 buffer supplemented with protease inhibitors (Roche). After 20 min in ice, cells were pelleted by centrifugation and the supernatant defined as the cytoplasmic fraction. After an additional wash in the extraction buffer, cell pellets were resuspended in 100 µL of a 20 mM Hepes pH 7.5, 400 mM NaCl, 1 mM EDTA supplemented with protease inhibitors. After 30 min in ice, samples were sonicated and cleared by centrifugation. Supernatants constitute the nuclear fraction. α-tubulin and phosho-Serine 10 histone H3 were used as cytoplasmic and nuclear protein control, respectively.

List of primers used to assess gene expression by qRT-PCR

ID	Name	Sequence
NM_000041	*APOE*	5'-TGGACAAGTCTGGGATCCTT-3'
		5'-CATCTTCCTGCCTGTGATTG-3'
NM_014298	*QPRT*	5'-GTCACCATGGACGCTGAA-3'
		5'-GAGCCAGCTGTCACCAG-3'
NM_000194.1	*HPRT1*	5'-TGACCTTGATTTATTTTGCATACC-3'
		5'-CGAGCAAGACGTTCAGTCCT-3'
NM_000474.3	*TWIST1*	5'-GGCTCAGCTACGCCTCTC-3'
		5'-CCTTCTCTGGAAACAATGACATCT-3'
NM_057179	*TWIST2*	5'-CATGTCCGCCTCCCACTA-3'
		5'-GCATCATTCAGAATCTCCTCCT-3'
NM_001128128.1	*ZEB1*	5'-AACTGCTGGGAGGATGACAC-3'
		5'-TCCTGCTTCATCTGCCTGA-3'
NM_014795.2	*ZEB2*	5'-AAGCCAGGGACAGATCAGC-3'
		5'-GCCACACTCTGTGCATTTGA-3'
NM_005985	*SNAI1*	5'-GCTGCAGGACTCTAATCCAGA-3'
		5'-ATCTCCGGAGGTGGGATG-3'
NM_003068	*SNAI2*	5'-TGGTTGCTTCAAGGACACAT-3'
		5'-GTTGCAGTGAGGGCAAGAA-3'
NM_011546	*Zeb1*	5'-GCCAGCAGTCATGATGAAAA-3'<br>5'-TATCACAATACGGGCAGGTG-3'
NM_015753	*Zeb2*	5'-CCAGAGGAAACAAGGATTTCAG-3'<br>5'-AGGCCTGACATGTAGTCTTGTG-3'

Supplementary references

1. Irizarry RA., Hobbs B., Collin F., Beazer-Barclay YD., Antonellis KJ., Scherf U., et al. Exploration, normalization, and summaries of high density oligonucleotide array probe level data. *Biostatistics* 2003;**4**(2):249–64. Doi: 10.1093/biostatistics/4.2.249.
2. Dai M., Wang P., Boyd AD., Kostov G., Athey B., Jones EG., et al. Evolving gene/transcript definitions significantly alter the interpretation of GeneChip data. *Nucleic Acids Res* 2005;**33**(20):e175. Doi: 10.1093/nar/gni179.
3. Sandberg R., Larsson O. Improved precision and accuracy for microarrays using updated probe set definitions. *BMC Bioinformatics* 2007;**8**:48. Doi: 10.1186/1471-2105-8-48.
4. Maglott D., Ostell J., Pruitt KD., Tatusova T. Entrez Gene: gene-centered information at NCBI. *Nucleic Acids Res* 2005;**33**(Database issue):D54-58. Doi: 10.1093/nar/gki031.
5. Reich M., Liefeld T., Gould J., Lerner J., Tamayo P., Mesirov JP. GenePattern 2.0. *Nat Genet* 2006;**38**(5):500–1. Doi: 10.1038/ng0506-500.

6. Barbie DA., Tamayo P., Boehm JS., Kim SY., Moody SE., Dunn IF., et al. Systematic RNA interference reveals that oncogenic KRAS-driven cancers require TBK1. *Nature* 2009;**462**(7269):108–12. Doi: 10.1038/nature08460.
7. Subramanian A., Tamayo P., Mootha VK., Mukherjee S., Ebert BL., Gillette MA., et al. Gene set enrichment analysis: a knowledge-based approach for interpreting genome-wide expression profiles. *Proc Natl Acad Sci U S A* 2005;**102**(43):15545–50. Doi: 10.1073/pnas.0506580102.
8. Yoshihara K., Shahmoradgoli M., Martínez E., Vegesna R., Kim H., Torres-Garcia W., et al. Inferring tumour purity and stromal and immune cell admixture from expression data. *Nat Commun* 2013;**4**:2612. Doi: 10.1038/ncomms3612.
9. Merlos-Suárez A., Barriga FM., Jung P., Iglesias M., Céspedes MV., Rossell D., et al. The intestinal stem cell signature identifies colorectal cancer stem cells and predicts disease relapse. *Cell Stem Cell* 2011;**8**(5):511–24. Doi: 10.1016/j.stem.2011.02.020.

Supplementary Figure legend

Figure S1:

A. Confirmation of the ectopic expression of murine Zeb transcripts by qRT-PCR

Analysis of the ectopic expression of murine *Zeb1* and *Zeb2* in transduced HCEC-hTERT cells. Left panel: ectopic expression was assessed by qRT-PCR (Ct values are presented). Student's t-test, mean and SD of one experiment performed in triplicates are shown. Right panels: analysis of ectopic ZEB1 or ZEB2 protein by Western blot. Immortalized human mammary epithelial cells transduced with *Zeb2* were used as a positive protein detection control (*).

B. NF-κB is aberrantly activated in parental HCEC-hTERT cells

Analysis of the subcellular distribution of p65 NF-κB (p65) in parental HCEC-hTERT cells as assessed by Western blot. The significant detection of p65 in the nuclear compartment indicates an elevated NF-κB activity level at the steady state. Distribution of α-tubulin and phospho-Serine 10 histone H3 (PSer10-H3) is used as internal controls of the quality of the cytoplasmic and nuclear fractionation. C: cytoplasmic compartment, N: nuclear compartment.

Figure S2. **Several OSAP-associated proteins contribute to cell adaptation to stress**

(**A**) Ectopic expression of *QPRT* or *APOE* facilitates the escape from chronic inflammation-induced (+ AMS, left panel) or chemical peroxide-induced (+ TBHP, right panel) senescence, as assessed in a crystal violet assay (top panels). (**B**) Analysis of ectopically produced QPRT

and APOE proteins in HCEC-hTERT cells transduced with *APOE* or *QPRT* respectively by Western-blot.

Table S1: Enriched GOBP pathways from OSAP down-regulated genes

Table S2: Enriched GOBP pathways from OSAP up-regulated genes

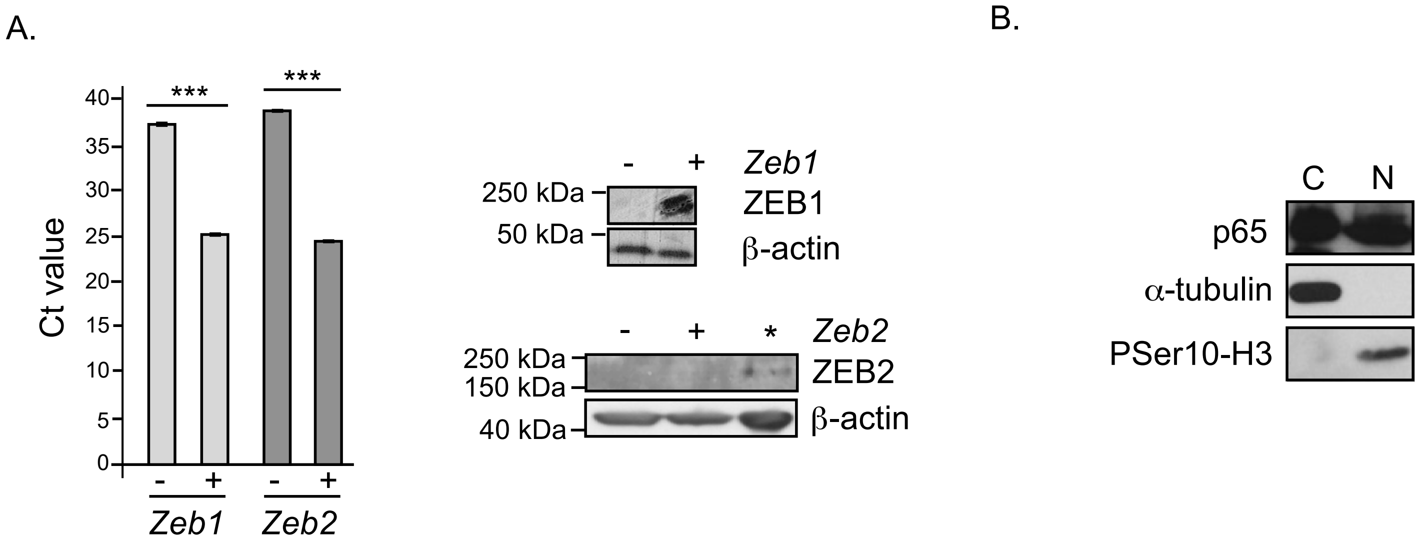
A.
B.
Ct value
40
35
30
25
20
15
10
5
0
***
***
- + - +
Zeb1
Zeb2
- + Zeb1
250 kDa
ZEB1
50 kDa
β-actin
- + * Zeb2
250 kDa
150 kDa
ZEB2
40 kDa
β-actin
C N
p65
α-tubulin
PSer10-H3

Figure S1

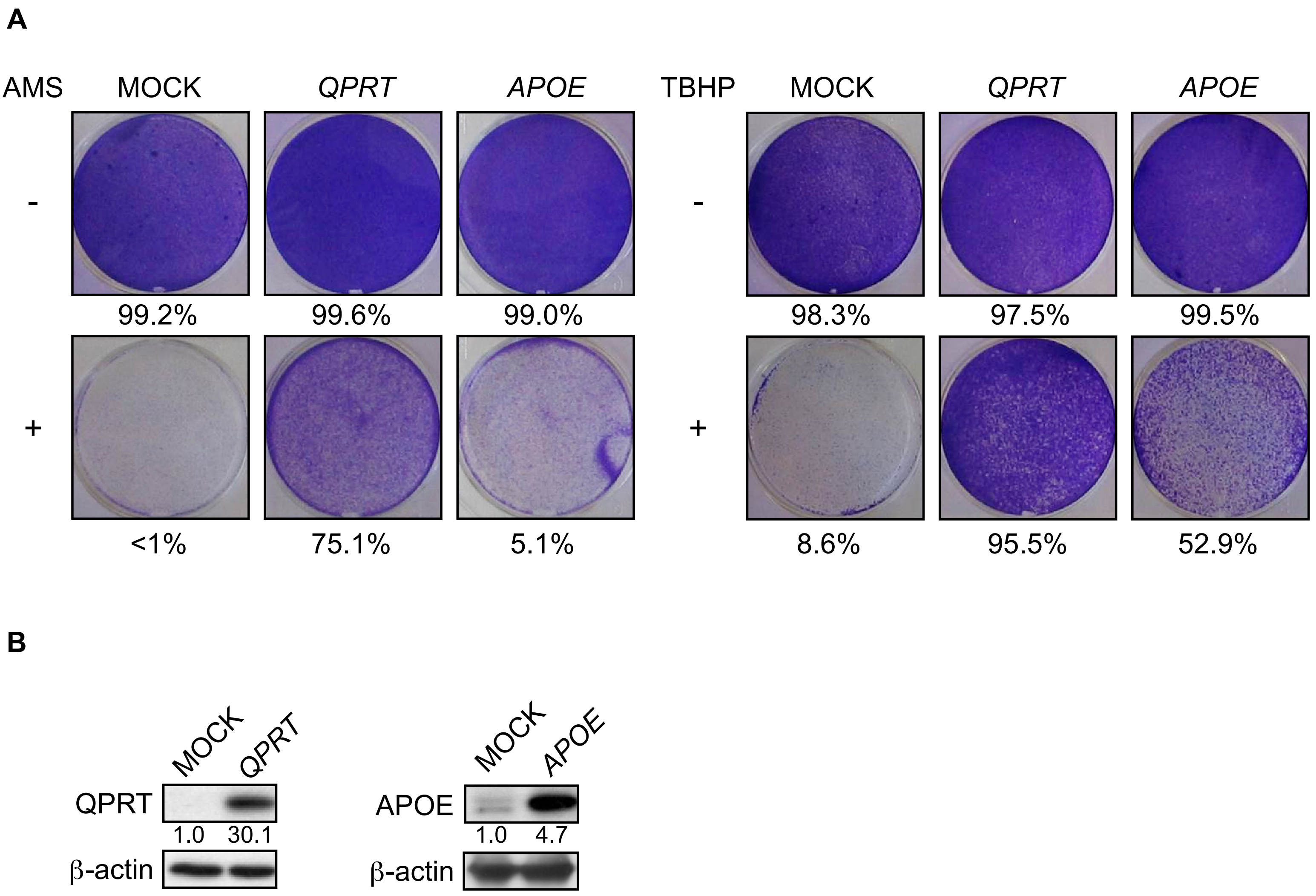
A
AMS
MOCK
QPRT
APOE
TBHP
MOCK
QPRT
APOE
-
+
99.2%
99.6%
99.0%
98.3%
97.5%
99.5%
<1%
75.1%
5.1%
8.6%
95.5%
52.9%
B
MOCK
QPRT
QPRT
1.0
30.1
β-actin
MOCK
APOE
APOE
1.0
4.7
β-actin

Figure S2

Table S1

	Description	BgRatio	p.adjust
GOBP_RESPONSE_TO_MOLECULE_OF_BACTERIAL_ORIGIN	GOBP_RESPONSE_TO_MOLECULE_OF_BACTERIAL_ORIGIN	354/17872	1.83E-06
GOBP_CYTOKINE_MEDIATED_SIGNALING_PATHWAY	GOBP_CYTOKINE_MEDIATED_SIGNALING_PATHWAY	485/17872	1.42E-05
GOBP_CELLULAR_RESPONSE_TO_MOLECULE_OF_BACTERIAL_ORIGIN	GOBP_CELLULAR_RESPONSE_TO_MOLECULE_OF_BACTERIAL_ORIGIN	229/17872	1.55E-05
GOBP_CELLULAR_RESPONSE_TO_BIOTIC_STIMULUS	GOBP_CELLULAR_RESPONSE_TO_BIOTIC_STIMULUS	256/17872	2.50E-05
GOBP_I_KAPPAB_KINASE_NF_KAPPAB_SIGNALING	GOBP_I_KAPPAB_KINASE_NF_KAPPAB_SIGNALING	288/17872	4.46E-05
GOBP_NEUTROPHIL_CHEMOTAXIS	GOBP_NEUTROPHIL_CHEMOTAXIS	105/17872	9.73E-05
GOBP_LEUKOCYTE_CHEMOTAXIS	GOBP_LEUKOCYTE_CHEMOTAXIS	236/17872	1.57E-04
GOBP_GRANULOCYTE_CHEMOTAXIS	GOBP_GRANULOCYTE_CHEMOTAXIS	127/17872	1.57E-04
GOBP_NEUTROPHIL_MIGRATION	GOBP_NEUTROPHIL_MIGRATION	128/17872	1.57E-04
GOBP_LEUKOCYTE_MIGRATION	GOBP_LEUKOCYTE_MIGRATION	385/17872	1.57E-04
GOBP_CHRONIC_INFLAMMATORY_RESPONSE	GOBP_CHRONIC_INFLAMMATORY_RESPONSE	17/17872	2.36E-04
GOBP_GRANULOCYTE_MIGRATION	GOBP_GRANULOCYTE_MIGRATION	154/17872	2.99E-04
GOBP_POSITIVE_REGULATION_OF_NF_KAPPAB_TRANSCRIPTION_FACTOR_ACTIVITY	GOBP_POSITIVE_REGULATION_OF_NF_KAPPAB_TRANSCRIPTION_FACTOR_ACTIVITY	154/17872	2.99E-04
GOBP_REGULATION_OF_DNA_BINDING_TRANSCRIPTION_FACTOR_ACTIVITY	GOBP_REGULATION_OF_DNA_BINDING_TRANSCRIPTION_FACTOR_ACTIVITY	451/17872	3.21E-04
GOBP_ANTIMICROBIAL_HUMORAL_IMMUNE_RESPONSE_MEDIATED_BY_ANTIMICROBIAL_PEPTIDE	GOBP_ANTIMICROBIAL_HUMORAL_IMMUNE_RESPONSE_MEDIATED_BY_ANTIMICROBIAL_PEPTID	79/17872	4.83E-04
GOBP_CELL_CHEMOTAXIS	GOBP_CELL_CHEMOTAXIS	315/17872	4.88E-04
GOBP_RESPONSE_TO_CHEMOKINE	GOBP_RESPONSE_TO_CHEMOKINE	96/17872	9.26E-04
GOBP_MYELOID_LEUKOCYTE_MIGRATION	GOBP_MYELOID_LEUKOCYTE_MIGRATION	230/17872	1.52E-03
GOBP_ANTIMICROBIAL_HUMORAL_RESPONSE	GOBP_ANTIMICROBIAL_HUMORAL_RESPONSE	123/17872	2.20E-03
GOBP_TOLL_LIKE_RECEPTOR_SIGNALING_PATHWAY	GOBP_TOLL_LIKE_RECEPTOR_SIGNALING_PATHWAY	126/17872	2.30E-03
GOBP_POSITIVE_REGULATION_OF_DNA_BINDING_TRANSCRIPTION_FACTOR_ACTIVITY	GOBP_POSITIVE_REGULATION_OF_DNA_BINDING_TRANSCRIPTION_FACTOR_ACTIVITY	264/17872	2.52E-03
GOBP_HUMORAL_IMMUNE_RESPONSE	GOBP_HUMORAL_IMMUNE_RESPONSE	312/17872	5.16E-03
GOBP_POSITIVE_REGULATION_OF_HEPATOCYTE_PROLIFERATION	GOBP_POSITIVE_REGULATION_OF_HEPATOCYTE_PROLIFERATION	10/17872	5.16E-03
GOBP_REGULATION_OF_CHRONIC_INFLAMMATORY_RESPONSE	GOBP_REGULATION_OF_CHRONIC_INFLAMMATORY_RESPONSE	10/17872	5.16E-03
GOBP_LIPOPOLYSACCHARIDE_MEDIATED_SIGNALING_PATHWAY	GOBP_LIPOPOLYSACCHARIDE_MEDIATED_SIGNALING_PATHWAY	62/17872	5.53E-03
GOBP_POSITIVE_REGULATION_OF_CELL_CELL_ADHESION_MEDIATED_BY_CADHERIN	GOBP_POSITIVE_REGULATION_OF_CELL_CELL_ADHESION_MEDIATED_BY_CADHERIN	11/17872	5.71E-03
GOBP_INTERLEUKIN_6_PRODUCTION	GOBP_INTERLEUKIN_6_PRODUCTION	172/17872	5.71E-03
GOBP_POSITIVE_REGULATION_OF_MONONUCLEAR_CELL_MIGRATION	GOBP_POSITIVE_REGULATION_OF_MONONUCLEAR_CELL_MIGRATION	66/17872	5.95E-03
GOBP_POSITIVE_REGULATION_OF_HAIR_FOLLICLE_DEVELOPMENT	GOBP_POSITIVE_REGULATION_OF_HAIR_FOLLICLE_DEVELOPMENT	12/17872	6.12E-03
GOBP_PATTERN_RECOGNITION_RECEPTOR_SIGNALING_PATHWAY	GOBP_PATTERN_RECOGNITION_RECEPTOR_SIGNALING_PATHWAY	180/17872	6.12E-03
GOBP_POSITIVE_REGULATION_OF_CHEMOKINE_PRODUCTION	GOBP_POSITIVE_REGULATION_OF_CHEMOKINE_PRODUCTION	70/17872	6.40E-03
GOBP_REGULATION_OF_NUCLEOTIDE_BINDING_OLIGOMERIZATION_DOMAIN_CONTAINING_SIGNALING_PATHWAY	GOBP_REGULATION_OF_NUCLEOTIDE_BINDING_OLIGOMERIZATION_DOMAIN_CONTAINING_SIGN	13/17872	6.58E-03
GOBP_POSITIVE_REGULATION_OF_I_KAPPAB_KINASE_NF_KAPPAB_SIGNALING	GOBP_POSITIVE_REGULATION_OF_I_KAPPAB_KINASE_NF_KAPPAB_SIGNALING	188/17872	6.58E-03
GOBP_REGULATION_OF_SECONDARY_METABOLIC_PROCESS	GOBP_REGULATION_OF_SECONDARY_METABOLIC_PROCESS	14/17872	7.13E-03
GOBP_REGULATION_OF_VASCULAR_WOUND_HEALING	GOBP_REGULATION_OF_VASCULAR_WOUND_HEALING	14/17872	7.13E-03
GOBP_REGULATION_OF_HEPATOCYTE_PROLIFERATION	GOBP_REGULATION_OF_HEPATOCYTE_PROLIFERATION	15/17872	7.99E-03
GOBP_L_GLUTAMATE_IMPORT_ACROSS_PLASMA_MEMBRANE	GOBP_L_GLUTAMATE_IMPORT_ACROSS_PLASMA_MEMBRANE	16/17872	8.80E-03
GOBP_RESPONSE_TO_VIRUS	GOBP_RESPONSE_TO_VIRUS	391/17872	8.80E-03
GOBP_REGULATION_OF_INFLAMMATORY_RESPONSE	GOBP_REGULATION_OF_INFLAMMATORY_RESPONSE	397/17872	9.20E-03
GOBP_LEUKOCYTE_MIGRATION_INVOLVED_IN_INFLAMMATORY_RESPONSE	GOBP_LEUKOCYTE_MIGRATION_INVOLVED_IN_INFLAMMATORY_RESPONSE	19/17872	1.11E-02
GOBP_REGULATION_OF_HAIR_FOLLICLE_DEVELOPMENT	GOBP_REGULATION_OF_HAIR_FOLLICLE_DEVELOPMENT	19/17872	1.11E-02
GOBP_REGULATION_OF_INNATE_IMMUNE_RESPONSE	GOBP_REGULATION_OF_INNATE_IMMUNE_RESPONSE	230/17872	1.11E-02
GOBP_REGULATION_OF_CYSTEINE_TYPE_ENDOPEPTIDASE_ACTIVITY	GOBP_REGULATION_OF_CYSTEINE_TYPE_ENDOPEPTIDASE_ACTIVITY	232/17872	1.11E-02
GOBP_POSITIVE_REGULATION_OF_JNK_CASCADE	GOBP_POSITIVE_REGULATION_OF_JNK_CASCADE	95/17872	1.11E-02
GOBP_RESPONSE_TO_MURAMYL_DIPEPTIDE	GOBP_RESPONSE_TO_MURAMYL_DIPEPTIDE	20/17872	1.11E-02
GOBP_TOLL_LIKE_RECEPTOR_3_SIGNALING_PATHWAY	GOBP_TOLL_LIKE_RECEPTOR_3_SIGNALING_PATHWAY	20/17872	1.11E-02
GOBP_POSITIVE_REGULATION_OF_INTERLEUKIN_6_PRODUCTION	GOBP_POSITIVE_REGULATION_OF_INTERLEUKIN_6_PRODUCTION	97/17872	1.11E-02
GOBP_CHEMOKINE_PRODUCTION	GOBP_CHEMOKINE_PRODUCTION	98/17872	1.12E-02
GOBP_REGULATION_OF_CELL_CELL_ADHESION_MEDIATED_BY_CADHERIN	GOBP_REGULATION_OF_CELL_CELL_ADHESION_MEDIATED_BY_CADHERIN	21/17872	1.17E-02
GOBP_RESPONSE_TO_TUMOR_NECROSIS_FACTOR	GOBP_RESPONSE_TO_TUMOR_NECROSIS_FACTOR	248/17872	1.23E-02
GOBP_VASCULAR_WOUND_HEALING	GOBP_VASCULAR_WOUND_HEALING	22/17872	1.23E-02
GOBP_LIVER_MORPHOGENESIS	GOBP_LIVER_MORPHOGENESIS	23/17872	1.31E-02
GOBP_TUMOR_NECROSIS_FACTOR_MEDIATED_SIGNALING_PATHWAY	GOBP_TUMOR_NECROSIS_FACTOR_MEDIATED_SIGNALING_PATHWAY	107/17872	1.31E-02
GOBP_SECONDARY_METABOLITE_BIOSYNTHETIC_PROCESS	GOBP_SECONDARY_METABOLITE_BIOSYNTHETIC_PROCESS	25/17872	1.51E-02
GOBP_MOLTING_CYCLE	GOBP_MOLTING_CYCLE	114/17872	1.52E-02
GOBP_REGULATION_OF_MONONUCLEAR_CELL_MIGRATION	GOBP_REGULATION_OF_MONONUCLEAR_CELL_MIGRATION	116/17872	1.55E-02
GOBP_NUCLEOTIDE_BINDING_DOMAIN_LEUCINE_RICH_REPEAT_CONTAINING_RECEPTOR_SIGNALING_PATHWAY	GOBP_NUCLEOTIDE_BINDING_DOMAIN_LEUCINE_RICH_REPEAT_CONTAINING_RECEPTOR_SIGNAL	26/17872	1.55E-02
GOBP_CELLULAR_RESPONSE_TO_INTERFERON_GAMMA	GOBP_CELLULAR_RESPONSE_TO_INTERFERON_GAMMA	117/17872	1.55E-02
GOBP_POSITIVE_REGULATION_OF_CYTOKINE_PRODUCTION_INVOLVED_IN_INFLAMMATORY_RESPONSE	GOBP_POSITIVE_REGULATION_OF_CYTOKINE_PRODUCTION_INVOLVED_IN_INFLAMMATORY_RES	27/17872	1.61E-02
GOBP_CELL_CELL_ADHESION_MEDIATED_BY_CADHERIN	GOBP_CELL_CELL_ADHESION_MEDIATED_BY_CADHERIN	28/17872	1.68E-02
GOBP_REGULATION_OF_HAIR_CYCLE	GOBP_REGULATION_OF_HAIR_CYCLE	28/17872	1.68E-02
GOBP_GLAND_MORPHOGENESIS	GOBP_GLAND_MORPHOGENESIS	123/17872	1.68E-02
GOBP_POSITIVE_REGULATION_OF_DEFENSE_RESPONSE	GOBP_POSITIVE_REGULATION_OF_DEFENSE_RESPONSE	288/17872	1.71E-02
GOBP_L_GLUTAMATE_TRANSMEMBRANE_TRANSPORT	GOBP_L_GLUTAMATE_TRANSMEMBRANE_TRANSPORT	29/17872	1.71E-02
GOBP_REGULATION_OF_EPITHELIAL_CELL_MIGRATION	GOBP_REGULATION_OF_EPITHELIAL_CELL_MIGRATION	291/17872	1.74E-02
GOBP_INTERLEUKIN_1_PRODUCTION	GOBP_INTERLEUKIN_1_PRODUCTION	130/17872	1.84E-02
GOBP_ANGIOGENESIS_INVOLVED_IN_WOUND_HEALING	GOBP_ANGIOGENESIS_INVOLVED_IN_WOUND_HEALING	31/17872	1.84E-02
GOBP_POSITIVE_REGULATION_OF_T_CELL_MIGRATION	GOBP_POSITIVE_REGULATION_OF_T_CELL_MIGRATION	31/17872	1.84E-02
GOBP_POSITIVE_REGULATION_OF_STRESS_ACTIVATED_PROTEIN_KINASE_SIGNALING_CASCADE	GOBP_POSITIVE_REGULATION_OF_STRESS_ACTIVATED_PROTEIN_KINASE_SIGNALING_CASCADE	133/17872	1.89E-02
GOBP_NEGATIVE_REGULATION_OF_CELL_JUNCTION_ASSEMBLY	GOBP_NEGATIVE_REGULATION_OF_CELL_JUNCTION_ASSEMBLY	32/17872	1.91E-02
GOBP_NEURON_DEATH_IN_RESPONSE_TO_OXIDATIVE_STRESS	GOBP_NEURON_DEATH_IN_RESPONSE_TO_OXIDATIVE_STRESS	33/17872	2.00E-02
GOBP_RESPONSE_TO_INTERFERON_GAMMA	GOBP_RESPONSE_TO_INTERFERON_GAMMA	139/17872	2.06E-02
GOBP_POSITIVE_REGULATION_OF_LEUKOCYTE_MIGRATION	GOBP_POSITIVE_REGULATION_OF_LEUKOCYTE_MIGRATION	141/17872	2.09E-02
GOBP_REGULATION_OF_JNK_CASCADE	GOBP_REGULATION_OF_JNK_CASCADE	141/17872	2.09E-02
GOBP_POSITIVE_REGULATION_OF_INFLAMMATORY_RESPONSE	GOBP_POSITIVE_REGULATION_OF_INFLAMMATORY_RESPONSE	145/17872	2.23E-02
GOBP_POSITIVE_REGULATION_OF_CYSTEINE_TYPE_ENDOPEPTIDASE_ACTIVITY	GOBP_POSITIVE_REGULATION_OF_CYSTEINE_TYPE_ENDOPEPTIDASE_ACTIVITY	146/17872	2.24E-02
GOBP_POSITIVE_REGULATION_OF_LYMPHOCYTE_MIGRATION	GOBP_POSITIVE_REGULATION_OF_LYMPHOCYTE_MIGRATION	37/17872	2.32E-02
GOBP_CELLULAR_RESPONSE_TO_CHEMICAL_STRESS	GOBP_CELLULAR_RESPONSE_TO_CHEMICAL_STRESS	334/17872	2.42E-02
GOBP_REGULATION_OF_RESPONSE_TO_BIOTIC_STIMULUS	GOBP_REGULATION_OF_RESPONSE_TO_BIOTIC_STIMULUS	350/17872	2.84E-02
GOBP_NEURON_DEATH	GOBP_NEURON_DEATH	352/17872	2.86E-02
GOBP_ASTROCYTE_DEVELOPMENT	GOBP_ASTROCYTE_DEVELOPMENT	43/17872	2.94E-02
GOBP_POSITIVE_REGULATION_OF_JUN_KINASE_ACTIVITY	GOBP_POSITIVE_REGULATION_OF_JUN_KINASE_ACTIVITY	43/17872	2.94E-02
GOBP_DEFENSE_RESPONSE_TO_BACTERIUM	GOBP_DEFENSE_RESPONSE_TO_BACTERIUM	361/17872	3.00E-02
GOBP_REGULATION_OF_T_CELL_MIGRATION	GOBP_REGULATION_OF_T_CELL_MIGRATION	44/17872	3.00E-02
GOBP_AMINO_ACID_IMPORT_ACROSS_PLASMA_MEMBRANE	GOBP_AMINO_ACID_IMPORT_ACROSS_PLASMA_MEMBRANE	45/17872	3.10E-02
GOBP_TISSUE_MIGRATION	GOBP_TISSUE_MIGRATION	368/17872	3.13E-02
GOBP_JNK_CASCADE	GOBP_JNK_CASCADE	175/17872	3.19E-02
GOBP_ACID_SECRETION	GOBP_ACID_SECRETION	47/17872	3.19E-02
GOBP_MEMBRANE_PROTEIN_ECTODOMAIN_PROTEOLYSIS	GOBP_MEMBRANE_PROTEIN_ECTODOMAIN_PROTEOLYSIS	47/17872	3.19E-02
GOBP_PHENOL_CONTAINING_COMPOUND_BIOSYNTHETIC_PROCESS	GOBP_PHENOL_CONTAINING_COMPOUND_BIOSYNTHETIC_PROCESS	47/17872	3.19E-02
GOBP_SENSORY_PERCEPTION_OF_MECHANICAL_STIMULUS	GOBP_SENSORY_PERCEPTION_OF_MECHANICAL_STIMULUS	177/17872	3.25E-02
GOBP_REGULATION_OF_RESPONSE_TO_CYTOKINE_STIMULUS	GOBP_REGULATION_OF_RESPONSE_TO_CYTOKINE_STIMULUS	178/17872	3.27E-02
GOBP_PROGRAMMED_NECROTIC_CELL_DEATH	GOBP_PROGRAMMED_NECROTIC_CELL_DEATH	49/17872	3.32E-02
GOBP_RESPONSE_TO_NICOTINE	GOBP_RESPONSE_TO_NICOTINE	49/17872	3.32E-02
GOBP_REGULATION_OF_MORPHOGENESIS_OF_A_BRANCHING_STRUCTURE	GOBP_REGULATION_OF_MORPHOGENESIS_OF_A_BRANCHING_STRUCTURE	51/17872	3.55E-02
GOBP_REGULATION_OF_ADAPTIVE_IMMUNE_RESPONSE	GOBP_REGULATION_OF_ADAPTIVE_IMMUNE_RESPONSE	188/17872	3.62E-02
GOBP_TUMOR_NECROSIS_FACTOR_SUPERFAMILY_CYTOKINE_PRODUCTION	GOBP_TUMOR_NECROSIS_FACTOR_SUPERFAMILY_CYTOKINE_PRODUCTION	188/17872	3.62E-02
GOBP_DEFENSE_RESPONSE_TO_FUNGUS	GOBP_DEFENSE_RESPONSE_TO_FUNGUS	53/17872	3.72E-02
GOBP_AMINO_ACID_IMPORT	GOBP_AMINO_ACID_IMPORT	54/17872	3.82E-02
GOBP_POSITIVE_REGULATION_OF_PEPTIDASE_ACTIVITY	GOBP_POSITIVE_REGULATION_OF_PEPTIDASE_ACTIVITY	195/17872	3.89E-02
GOBP_MONONUCLEAR_CELL_MIGRATION	GOBP_MONONUCLEAR_CELL_MIGRATION	199/17872	4.08E-02
GOBP_MEMBRANE_PROTEIN_PROTEOLYSIS	GOBP_MEMBRANE_PROTEIN_PROTEOLYSIS	58/17872	4.19E-02
GOBP_REGULATION_OF_STRESS_ACTIVATED_PROTEIN_KINASE_SIGNALING_CASCADE	GOBP_REGULATION_OF_STRESS_ACTIVATED_PROTEIN_KINASE_SIGNALING_CASCADE	203/17872	4.19E-02
GOBP_RESPIRATORY_SYSTEM_DEVELOPMENT	GOBP_RESPIRATORY_SYSTEM_DEVELOPMENT	203/17872	4.19E-02
GOBP_NEGATIVE_REGULATION_OF_FAT_CELL_DIFFERENTIATION	GOBP_NEGATIVE_REGULATION_OF_FAT_CELL_DIFFERENTIATION	59/17872	4.20E-02
GOBP_POSITIVE_REGULATION_OF_NUCLEAR_DIVISION	GOBP_POSITIVE_REGULATION_OF_NUCLEAR_DIVISION	59/17872	4.20E-02
GOBP_SECONDARY_METABOLIC_PROCESS	GOBP_SECONDARY_METABOLIC_PROCESS	59/17872	4.20E-02
GOBP_REGULATION_OF_JUN_KINASE_ACTIVITY	GOBP_REGULATION_OF_JUN_KINASE_ACTIVITY	60/17872	4.30E-02
GOBP_POSITIVE_REGULATION_OF_EPITHELIAL_CELL_PROLIFERATION	GOBP_POSITIVE_REGULATION_OF_EPITHELIAL_CELL_PROLIFERATION	209/17872	4.30E-02
GOBP_WOUND_HEALING	GOBP_WOUND_HEALING	431/17872	4.30E-02
GOBP_CYTOPLASMIC_PATTERN_RECOGNITION_RECEPTOR_SIGNALING_PATHWAY	GOBP_CYTOPLASMIC_PATTERN_RECOGNITION_RECEPTOR_SIGNALING_PATHWAY	61/17872	4.30E-02
GOBP_RESPONSE_TO_OXIDATIVE_STRESS	GOBP_RESPONSE_TO_OXIDATIVE_STRESS	436/17872	4.30E-02
GOBP_POSITIVE_REGULATION_OF_INTERLEUKIN_1_BETA_PRODUCTION	GOBP_POSITIVE_REGULATION_OF_INTERLEUKIN_1_BETA_PRODUCTION	62/17872	4.30E-02
GOBP_POSITIVE_REGULATION_OF_INTERLEUKIN_8_PRODUCTION	GOBP_POSITIVE_REGULATION_OF_INTERLEUKIN_8_PRODUCTION	62/17872	4.30E-02
GOBP_REGULATION_OF_SYNAPSE_STRUCTURE_OR_ACTIVITY	GOBP_REGULATION_OF_SYNAPSE_STRUCTURE_OR_ACTIVITY	213/17872	4.30E-02
GOBP_ACIDIC_AMINO_ACID_TRANSPORT	GOBP_ACIDIC_AMINO_ACID_TRANSPORT	63/17872	4.30E-02
GOBP_INTERLEUKIN_2_PRODUCTION	GOBP_INTERLEUKIN_2_PRODUCTION	63/17872	4.30E-02
GOBP_LYMPHOCYTE_CHEMOTAXIS	GOBP_LYMPHOCYTE_CHEMOTAXIS	63/17872	4.30E-02
GOBP_REGULATION_OF_LYMPHOCYTE_MIGRATION	GOBP_REGULATION_OF_LYMPHOCYTE_MIGRATION	63/17872	4.30E-02
GOBP_POSITIVE_REGULATION_OF_RESPONSE_TO_EXTERNAL_STIMULUS	GOBP_POSITIVE_REGULATION_OF_RESPONSE_TO_EXTERNAL_STIMULUS	441/17872	4.30E-02
GOBP_RESPONSE_TO_FUNGUS	GOBP_RESPONSE_TO_FUNGUS	64/17872	4.36E-02
GOBP_POSITIVE_REGULATION_OF_CELL_ADHESION	GOBP_POSITIVE_REGULATION_OF_CELL_ADHESION	446/17872	4.38E-02
GOBP_REGULATION_OF_LEUKOCYTE_MIGRATION	GOBP_REGULATION_OF_LEUKOCYTE_MIGRATION	219/17872	4.38E-02
GOBP_REGULATION_OF_MORPHOGENESIS_OF_AN_EPITHELIUM	GOBP_REGULATION_OF_MORPHOGENESIS_OF_AN_EPITHELIUM	65/17872	4.38E-02
GOBP_POSITIVE_REGULATION_OF_ERK1_AND_ERK2_CASCADE	GOBP_POSITIVE_REGULATION_OF_ERK1_AND_ERK2_CASCADE	223/17872	4.44E-02
GOBP_CELLULAR_RESPONSE_TO_RETINOIC_ACID	GOBP_CELLULAR_RESPONSE_TO_RETINOIC_ACID	67/17872	4.44E-02
GOBP_ENDOTHELIAL_CELL_APOPTOTIC_PROCESS	GOBP_ENDOTHELIAL_CELL_APOPTOTIC_PROCESS	67/17872	4.44E-02
GOBP_L_ALPHA_AMINO_ACID_TRANSMEMBRANE_TRANSPORT	GOBP_L_ALPHA_AMINO_ACID_TRANSMEMBRANE_TRANSPORT	67/17872	4.44E-02
GOBP_NECROTIC_CELL_DEATH	GOBP_NECROTIC_CELL_DEATH	67/17872	4.44E-02
GOBP_T_CELL_MIGRATION	GOBP_T_CELL_MIGRATION	67/17872	4.44E-02
GOBP_REGULATION_OF_PEPTIDASE_ACTIVITY	GOBP_REGULATION_OF_PEPTIDASE_ACTIVITY	457/17872	4.46E-02
GOBP_AMYLOID_PRECURSOR_PROTEIN_CATABOLIC_PROCESS	GOBP_AMYLOID_PRECURSOR_PROTEIN_CATABOLIC_PROCESS	68/17872	4.46E-02
GOBP_PIGMENT_BIOSYNTHETIC_PROCESS	GOBP_PIGMENT_BIOSYNTHETIC_PROCESS	68/17872	4.46E-02
GOBP_L_AMINO_ACID_TRANSPORT	GOBP_L_AMINO_ACID_TRANSPORT	69/17872	4.53E-02
GOBP_NEGATIVE_REGULATION_OF_INTERLEUKIN_6_PRODUCTION	GOBP_NEGATIVE_REGULATION_OF_INTERLEUKIN_6_PRODUCTION	69/17872	4.53E-02
GOBP_REGULATION_OF_ENDOTHELIAL_CELL_MIGRATION	GOBP_REGULATION_OF_ENDOTHELIAL_CELL_MIGRATION	231/17872	4.60E-02
GOBP_POSITIVE_REGULATION_OF_PROTEIN_CATABOLIC_PROCESS	GOBP_POSITIVE_REGULATION_OF_PROTEIN_CATABOLIC_PROCESS	234/17872	4.73E-02
GOBP_POSITIVE_REGULATION_OF_INTERLEUKIN_1_PRODUCTION	GOBP_POSITIVE_REGULATION_OF_INTERLEUKIN_1_PRODUCTION	73/17872	4.94E-02
GOBP_IMMUNE_RESPONSE_REGULATING_SIGNALING_PATHWAY	GOBP_IMMUNE_RESPONSE_REGULATING_SIGNALING_PATHWAY	479/17872	4.97E-02

Table S2

	Description	BgRatio	p.adjust
GOBP_VASCULAR_PROCESS_IN_CIRCULATORY_SYSTEM	GOBP_VASCULAR_PROCESS_IN_CIRCULATORY_SYSTEM	263/17872	1.34E-02
GOBP_REGULATION_OF_VASCULAR_PERMEABILITY	GOBP_REGULATION_OF_VASCULAR_PERMEABILITY	48/17872	1.34E-02
GOBP_REGULATION_OF_SKELETAL_MUSCLE_ADAPTATION	GOBP_REGULATION_OF_SKELETAL_MUSCLE_ADAPTATION	14/17872	3.68E-02
GOBP_VASCULAR_TRANSPORT	GOBP_VASCULAR_TRANSPORT	87/17872	3.68E-02
GOBP_POSITIVE_REGULATION_OF_VASCULAR_PERMEABILITY	GOBP_POSITIVE_REGULATION_OF_VASCULAR_PERMEABILITY	17/17872	3.68E-02
