# Supplementary material for: Intestinal Epithelial Cells Adapt to Chronic Inflammation through Partial Genetic Reprogramming
Source: Cancers (Basel). 2023 Feb 3;15(3):973. doi: 10.3390/cancers15030973 (PMC9913703; doi:10.3390/cancers15030973)
Supplement: Supplementary file 1 [file cancers-15-00973-s001.zip › cancers-2188031-Supplementary File S2.pdf]

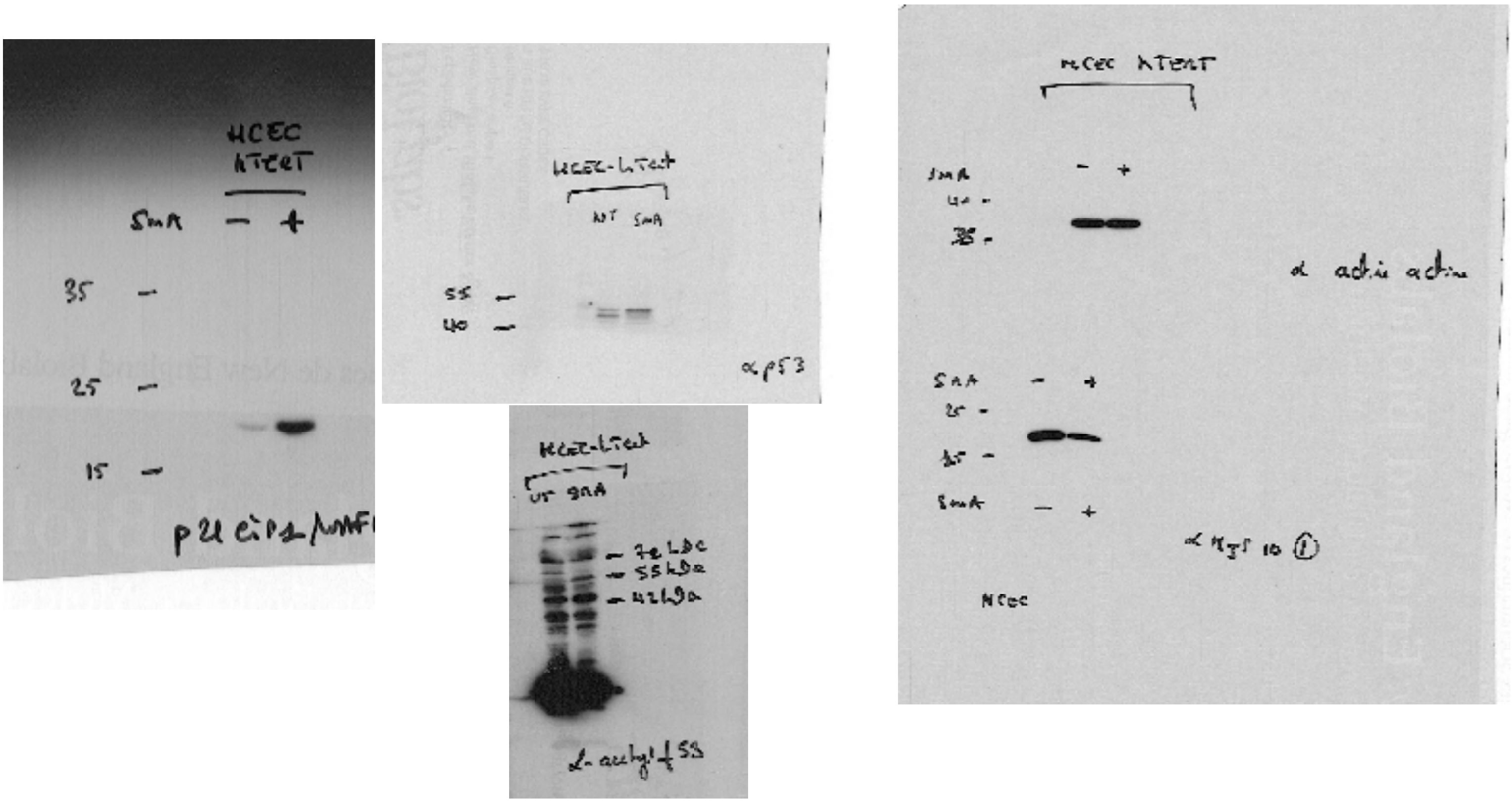

Figure 1B left panels : Western p53, p21, actin, Phospho S10-H3 and acetyl p53

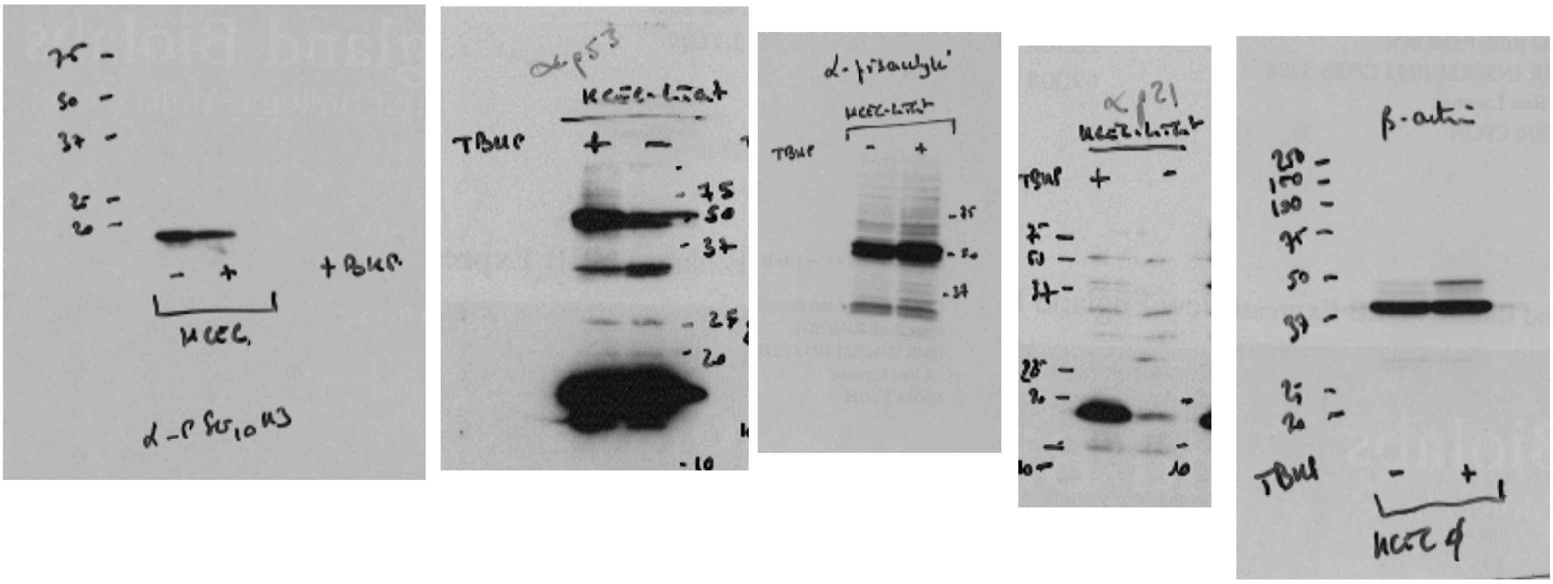

Figure 1B right panels : Western p53, p21, actin, Phospho S10-H3 and acetyl p53

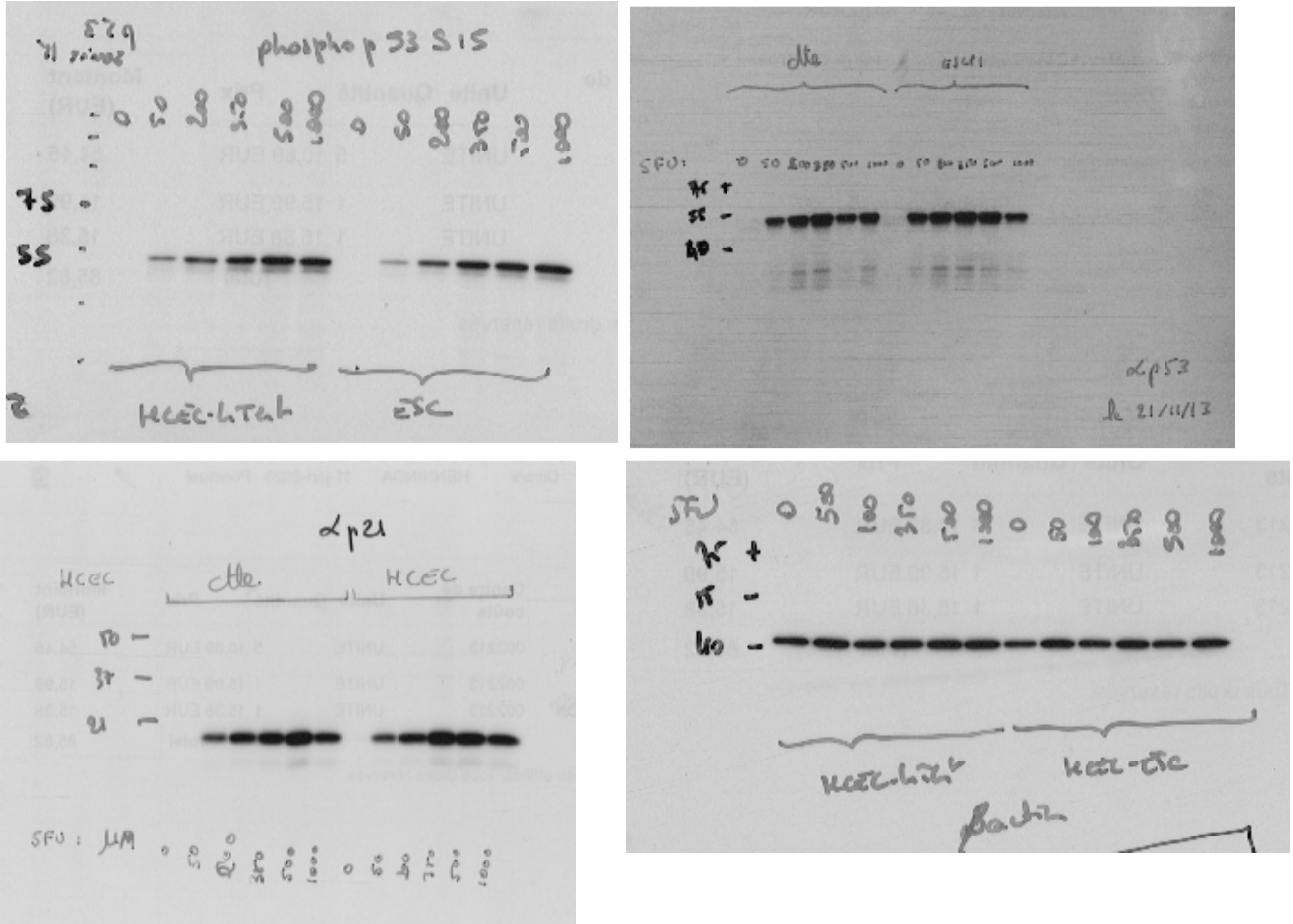

Figure 2 Western de phospho p53 S15, p53, p21 et actin

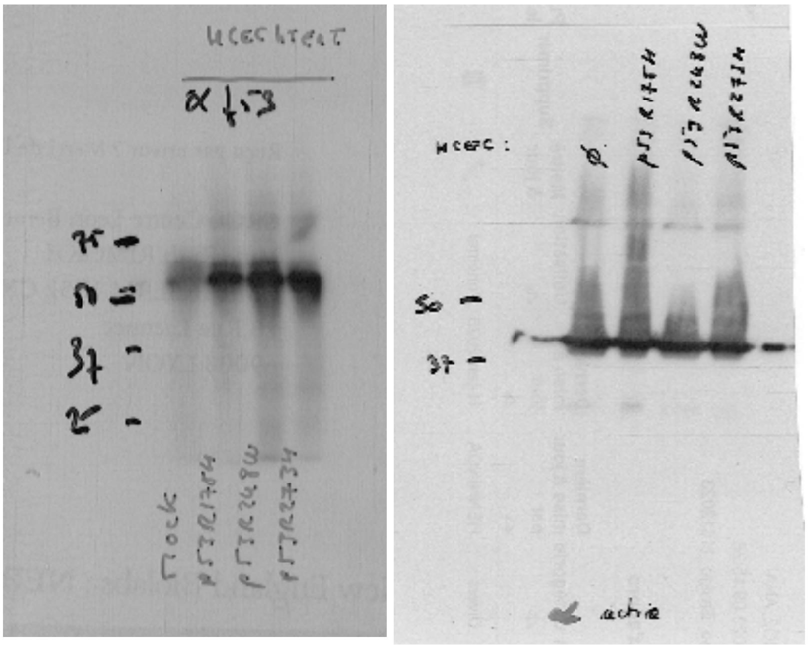

Figure 2 Expression control of p53 mutants

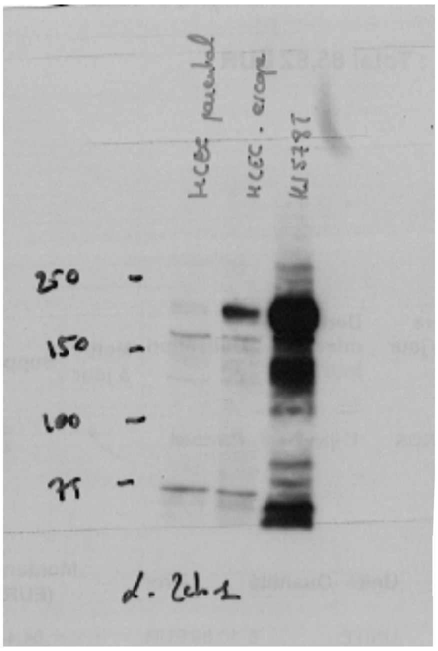

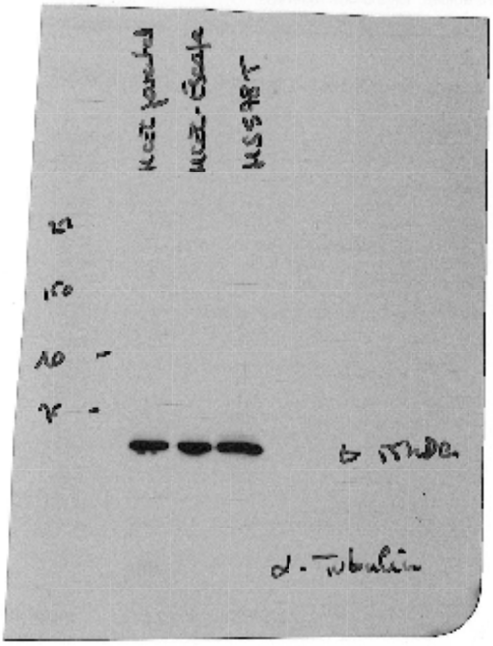

Figure 3 Analysis of ZEB1 expression

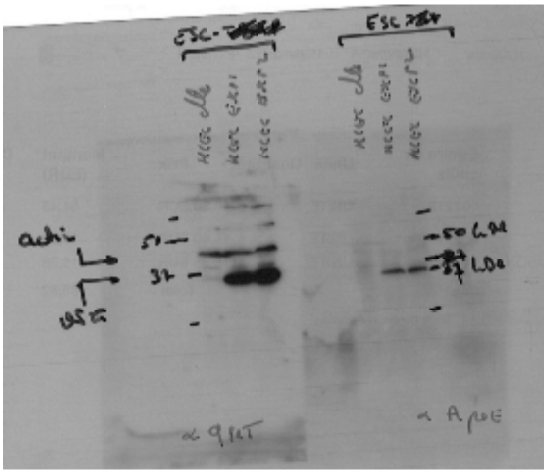

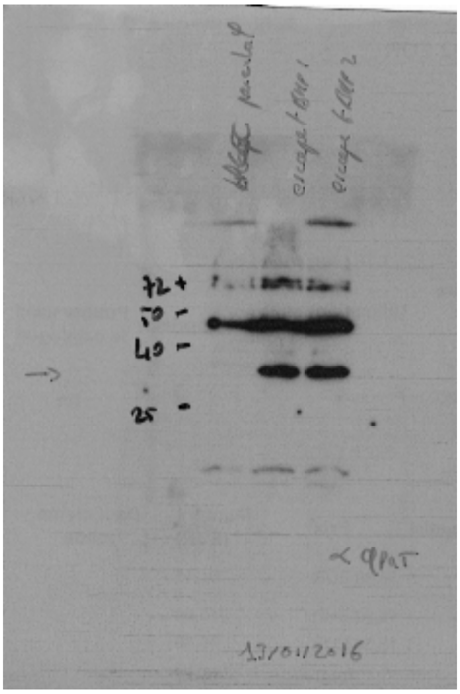

Figure 4C upper panels

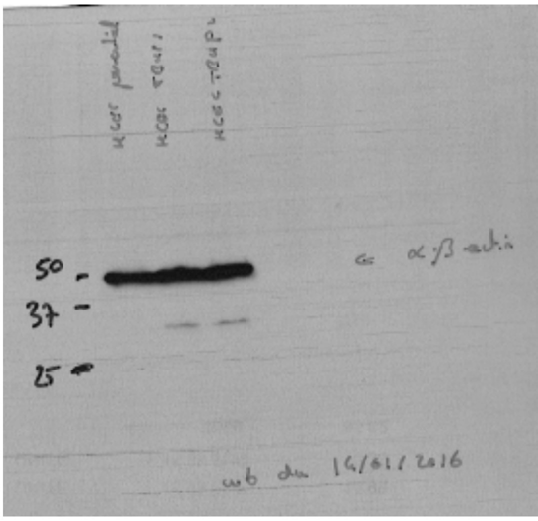

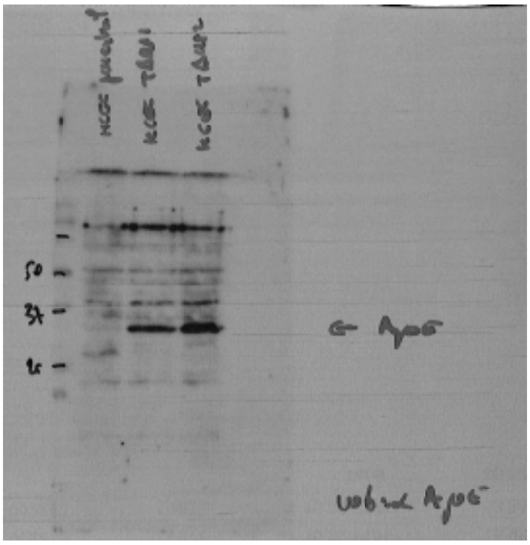

Figure 4C lower panels

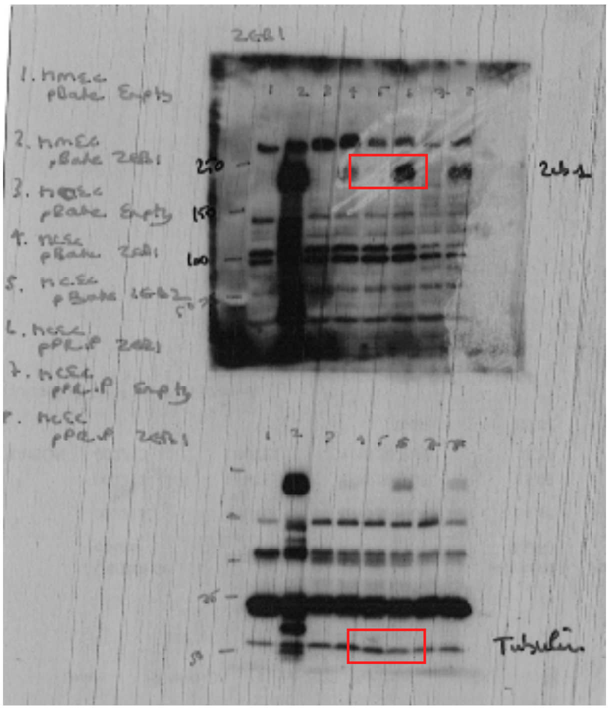

Figure S1 top left panel

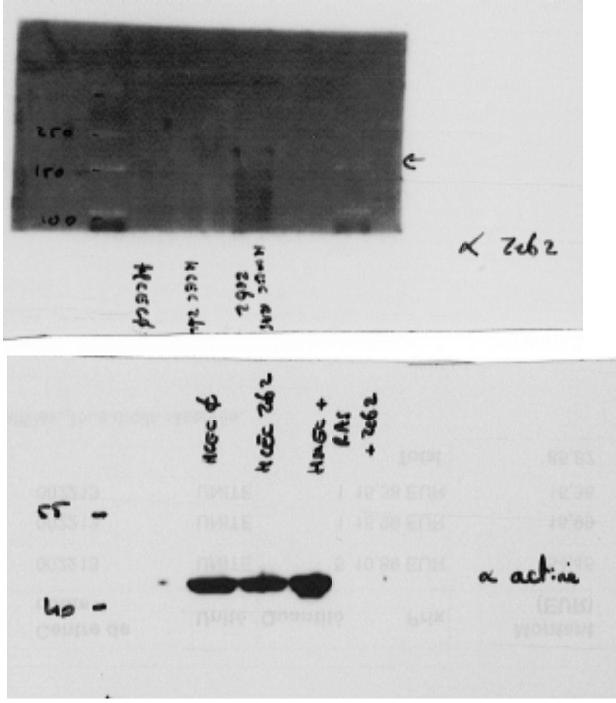

Figure S1 lower left panel

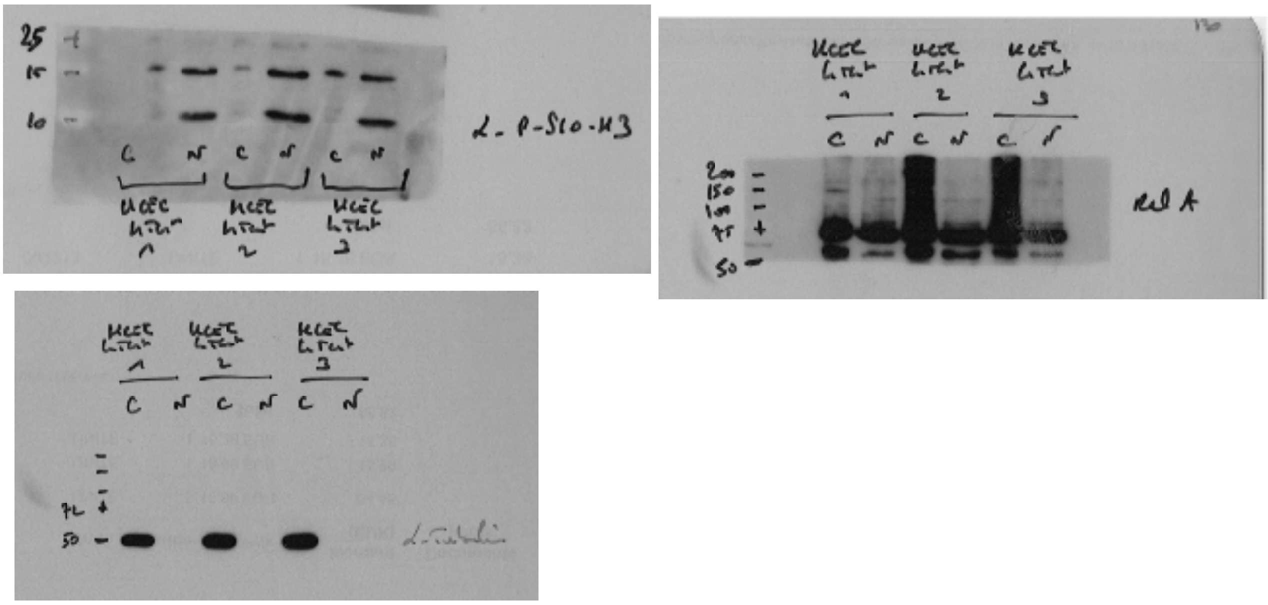

Figure S1 right panels

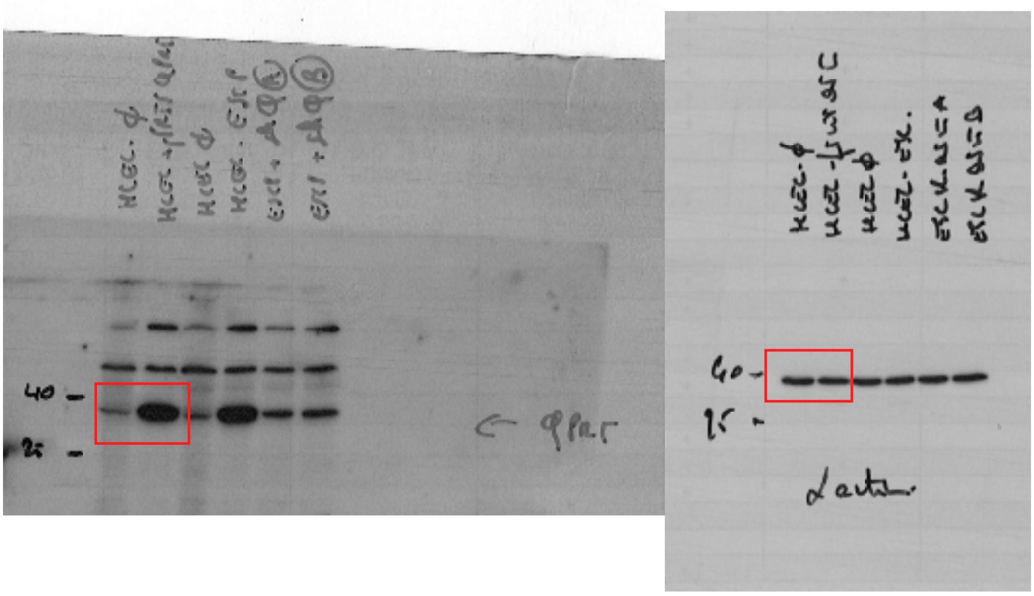

Figure S2 lefts panels

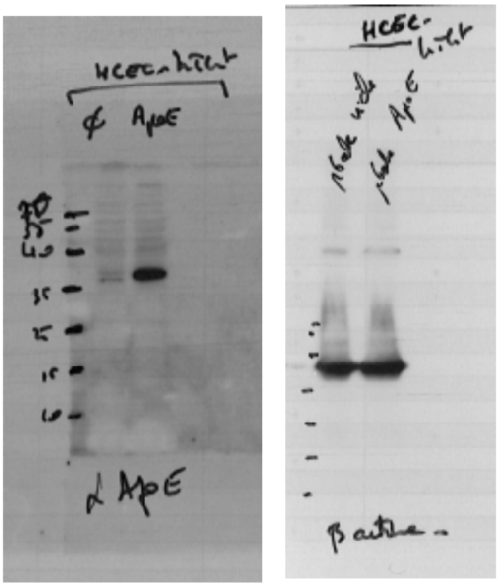

Figure S2 right panels
